# How to Conduct International Geriatric Rehabilitation Research?

**DOI:** 10.3390/jcm12030951

**Published:** 2023-01-26

**Authors:** Miriam L. Haaksma, Adam L. Gordon, Eléonore F. van Dam van Isselt, Jos M. G. A. Schols, Irma H. J. Everink, Ian D. Cameron, Clemens Becker, Stefan Grund, Wilco P. Achterberg

**Affiliations:** 1 University Network for the Care Sector South-Holland, Leiden University Medical Center, 2333 ZA Leiden, The Netherlands; 2 Department of Public Health and Primary Care, Leiden University Medical Center, 2333 ZA Leiden, The Netherlands; 3 School of Medicine, University of Nottingham, Nottingham NG7 2UH, UK; 4 Department of Health Services Research, Care and Public Health Research Institute, Maastricht University, 6200 MD Maastricht, The Netherlands; 5 John Walsh Centre for Rehabilitation Research, Northern Sydney Local Health District and Faculty of Medicine and Health, University of Sydney, Sydney, NSW 2006, Australia; 6 Department of Clinical Gerontology and Geriatric Rehabilitation, Robert Bosch Hospital, 70376 Stuttgart, Germany; 7 Center for Geriatric Medicine, Agaplesion Bethanien Hospital Heidelberg, Geriatric Center at the Heidelberg University, 69117 Heidelberg, Germany

**Keywords:** geriatric rehabilitation, study design, methodology

## Abstract

With an ageing global population and an increasing focus on aging in place, the number of people in need of geriatric rehabilitation (GR) is rapidly increasing. As current GR practice is very heterogenous, cross-country comparisons could allow us to learn from each other and optimise the effectiveness of GR. However, international GR research comes with many challenges. This article summarises the facilitators and barriers relating to the recruitment of rehabilitation centres, the inclusion of patients, and data collection, as experienced by experts in the field of international GR research. The three most important methodological recommendations for conducting cross-national collaborative research in the field of GR are (1) make use of existing (inter)national networks and social media to aid recruitment of GR centres; (2) clearly define the GR treatment, setting, and patient characteristics in the inclusion criteria; and (3) use a hierarchical study structure to communicate transparently and regularly with both national and local coordinators. International GR research would greatly benefit from the implementation of a core dataset in regular GR care. Therefore, future studies should focus on developing an international consensus regarding the outcomes and corresponding cross-culturally validated measurement instruments to be used during GR.

## 1. Introduction

With an ageing global population and increased focus on aging in place, the number of people who need geriatric rehabilitation (GR) is rapidly increasing [1]. In 2020, the GR special interest group of the European Geriatric Medicine Society (EuGMS) published a consensus statement on core principles and future priorities for GR [2]. This called for research to provide uniform treatment standards and international guidelines in order to standardise treatment quality in GR. As current GR practice is heterogenous, cross-country comparisons could help us learn from each other to optimise effectiveness of GR. Moreover, international studies into the effectiveness of specific treatments or innovations could enhance the generalizability of research results more than single-country studies. However, international GR research comes with many challenges.

In a recent pan-European study on recovery of post-COVID-19 patients in GR, over 700 patients from 59 health centres across 10 countries were recruited within 1 year [3]. This is a fine achievement, raising questions about why the recruitment was so efficient, which challenges were faced, and how they were overcome. During this COVID study—and other previous international GR studies—numerous facilitators and barriers for successful international GR research were encountered. Knowledge of these factors may contribute to the future success of pivotal international studies in the field. This article summarises facilitators and barriers to recruitment of rehabilitation centres, inclusion of patients, and data collection, as experienced by experts in GR research.

## 2. Facilitators and Barriers

An overview of the facilitators and barriers as experienced by the authors—i.e., a group of experts in GR research from four countries—is shown in Table 1.

### 2.1. Recruitment of GR Centres

Many geriatric rehabilitation centres can be reached through regional, national, and international networks. The largest international GR network is the GR special interest group of the EuGMS, which includes 20 member states. Using this network may prove beneficial to recruitment of GR centres. Using websites, newsletters, and social media channels of national geriatric societies can also support rapid recruitment. Publicity can rapidly spread around the world through social media channels of experts in the field as well. Recruitment of GR centres can also be facilitated by living labs. These are regional networks of healthcare facilities that are committed to improving care through research [4].

Rapidly publishing a protocol paper in an international peer-reviewed journal and registering the study (e.g., in a trial registry) can help inform the international research and practice community about a study’s aim, the required time investment, and the types of centres that are eligible to participate. The latter is particularly important, as the definition of a GR centre greatly differs between countries.

Although a consensus statement on the definition of GR is available [2], the setting (ambulatory, hospital-based, or nursing home based), team structure, therapies, treatment intensity, and length of stay are heterogeneous, both within and between countries [5]. These differences hamper a comparison of the effects of GR between countries. This means that, even when therapies are protocolised and standardised across centres, for example, in the context of an international RCT, there could still be issues with recruiting comparable populations in each centre, as triage criteria for GR differ between countries (e.g., in terms of age or comorbid conditions) due to different systems of care and health insurance policies. Therefore, it is pivotal to clearly define inclusion criteria relating to the population of interest, the rehabilitation setting, and the type of therapies patients are expected to receive, prior to the start of centre recruitment.

Another barrier for the recruitment of GR centres is the inconsistency of legislation and procedures relating to ethical approval across countries. While some countries may allow for an opt-out procedure in studies using pseudonymised regular care data, others may require informed consent. Each country has its own procedures which must be followed, including forms which must be filled out, in order to obtain ethical approval. These procedures and forms may even differ between institutions within the same country. Moreover, some specific paperwork, such as the patient information sheets, must be written in the local lay terms, which requires a translator who understands how to write for a lay audience.

**Figure 1:**
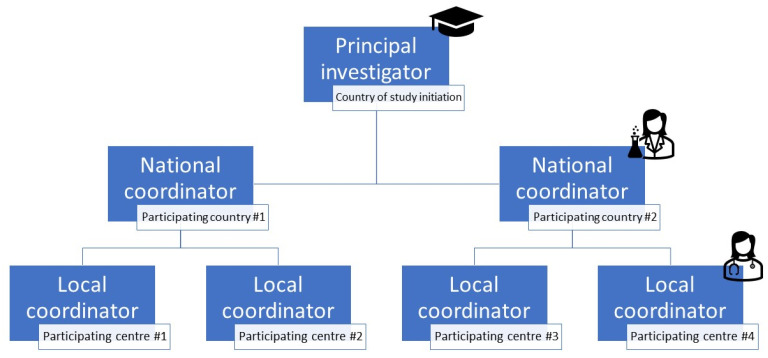
Example of a hierarchical study structure for a study in two countries with two participating centres each.

A hierarchical study structure, as shown in Figure 1, may facilitate the recruitment of centres. In this structure, the principal investigator (PI) appoints a national coordinator in each participating country who is responsible for the dissemination of study-related information among the local coordinators of the participating GR centres within their country. Ideally, these national coordinators have a strong network in the field of GR, as well as knowledge of national ethical regulations, in order to aid the start-up of the study in their country.

To further optimise the recruitment of GR centres, the research topic should be of interest to researchers in multiple countries and align with international societal needs. This will increase the motivation of all participating centres and their employees, which is particularly important when financial resources of the study are limited. For example, the urgency of the COVID-19 pandemic contributed to successful recruitment of GR centres in the EU-COGER study [3]. A physical kick-off meeting with all national coordinators can also increase motivation.

Last but not least, an important facilitator to recruitment of GR centres is transparency regarding future data sharing and (consortium) co-authorship. An offer of co-authorship may help to recruit centres, but the ICJME criteria for authorship must be adhered to and therefore the obligations of each contributor to secure authorship must be clear at the outset. The PI should draft a data sharing policy and a co-authorship policy in this regard. An example of a data sharing policy is shown in Supplementary Figure S1.

### 2.2. Inclusion of GR Patients

After selecting and recruiting suitable GR centres and obtaining the required ethical approval, the inclusion of GR patients can begin. Again, a hierarchical study structure (Figure 1) may be helpful. In this structure, one local coordinator is appointed in each participating centre who is responsible for the inclusion of patients and the communication of study information to all those involved within their centre. Ideally, local coordinators have a strong network within their GR centre as well as access to the (electronic) health records of patients to facilitate patient selection. Preferably, a back-up representative for each local coordinator is also appointed, who can take over their role when the original local coordinator changes jobs or falls ill. One-on-one start-up videocalls with each local coordinator and the PI can be very effective to communicate study procedures and clarify the inclusion criteria, as are written standard operating procedures (SOPs).

Even when inclusion criteria are clearly communicated in advance, they may be difficult to adhere to because the main diagnosis (or main reason for referral to GR) can be ambiguous for GR patients; they often suffer from multimorbidity. Questions from local coordinators about the eligibility of these ambiguous cases are therefore to be expected and the PI should be able to answer those questions promptly to facilitate inclusion. Ideally, the PI would have regular (i.e., at least monthly) videocalls with the national coordinators, and national coordinators would have regular videocalls or face-to-face meetings with local coordinators to keep everyone informed about the inclusions rates. Sharing preliminary results may have a motivating effect on local coordinators and thus facilitate inclusion.

As some GR centres may be recruited later than others, a flexible approach regarding prospective and retrospective recruitment of patients may improve inclusion rates as well. Of course, this is only possible when the study design allows for retrospective recruitment. In some (observational) studies, retrospective recruitment may be feasible. In that case, GR centres that were recruited relatively late may be allowed to recruit patients retrospectively, up to the start date of the study. Retrospective inclusion of patients who entered GR prior to the start of the study should be avoided, as this could introduce a cohort effect in the data [6]. Therefore, the range of eligible GR admission dates should be clearly specified in the inclusion criteria.

Language may also present a barrier for the inclusion of GR patients. A well-illustrated patient information sheet that is understandable for older people can facilitate inclusion. Moreover, some older patients—particularly first-generation migrants—may not be proficient in the local native language and may therefore not be able to fully understand the study information. For the representativeness of the study population, it is very important to include this group, as older immigrants who do not speak the local language are known to be at higher risk for poor physical and mental health outcomes compared to those who do speak the local language [7]. This means that patient information sheets, consent forms, and questionnaires must also be available in prominent minority languages for participating countries. It is therefore important to allow sufficient resources for translation, and to liaise with national coordinators about which languages translation is required for, and whether there are any national guidelines on how to support inclusion of ethnic minorities in research. Choosing measurement instruments that are available in multiple languages and are cross-culturally validated, and providing local coordinators with multiple versions of these instruments is highly recommended. An example of a questionnaire that is available in over 200 languages is the EQ-5D for measuring health-related quality of life [8]. For cognitive screening, the Rowland universal dementia assessment scale (RUDAS) is an example of a cross-culturally validated questionnaire with minimal need for language adaptation [9]. Unfortunately, not all cross-culturally validated measurement instruments are equally sensitive and specific for GR.

### 2.3. Data Collection

After recruitment of GR centres and the inclusion of patients, data collection can commence. Responsible data management requires good research documentation. It is therefore recommended to write a data management plan to ensure adherence to the FAIR data principles [10]. It is also useful to summarise the general project information (e.g., people involved, their roles, etc.), methodological information (e.g., methods of data collection and instruments), and data-specific information (e.g., variable names, units of measurement, data storage locations, etc.) in a metadata file, preferably written in English to ensure all international collaborators can read it.

Basing a study on regular care data from (electronic) health records has the advantage that no additional measurements need to be conducted, saving both time and money and facilitating data collection. However, a disadvantage of regular care data is that it differs considerably between countries. Both the time points at which patients are measured and the instruments used to measure them can differ between counties. The only functional outcome measure that is routinely collected across countries is the Barthel index [11]. Comparable measures are used in some countries, such as the Functional independence measure (FIM) [12] or the Utrecht scale for evaluation of rehabilitation (USER) [13], which can be converted to the Barthel index [14,15]. However, other outcome measures are not routinely collected across countries. Based on the International Classification of Functioning, Disability and Health (ICF), the World Health Organization developed a Geriatric ICF core set: a comprehensive and valid set of 29 ICF categories, reflecting health-related problems in older adults [16]. However, it does not list measurement instruments to be used within these categories. This speaks to the lack of international consensus on a core set of outcomes and corresponding measurement instruments to be used in GR, which greatly hampers research based on regular care data.

Collecting research data may be facilitated by using a GDPR- and/or HIPAA-compliant electronic data capture system such as REDCap [17] or Castor [18]. These systems allow local coordinators to easily enter research data through an online platform and ensure that the data is audit logged, securely stored, and regularly backed up. Theoretically, these systems can also facilitate automated data extraction from the electronic health records (EHR). However, this feature for automated data extraction needs to be integrated in the EHR system of each participating centre, which is hard given the large heterogeneity in EHR systems that are being used. EHR systems not only differ between countries, but also between centres within the same country, making large-scale automated data extraction unfeasible. Research data thus has to be manually copied to a research database by local coordinators or other professionals. The research field would benefit tremendously from more homogeneous EHR systems within and across countries.

In addition, entering research data may be facilitated by distributing clearly written SOPs among local coordinators. Ideally, these SOPs include the study’s inclusion criteria, as well as step-by-step instructions on what, how and when to measure patients, and which cut-offs to use for each measure. SOPs should also include instructions on how to login to the data capture system, how to enter the data, and, if applicable, how to store directly identifiable patient information in a separate key file.

## 3. Conclusions

The reported facilitators and barriers may prove useful for future cross-national collaborative research in the field of GR. However, it should be noted that these factors were not identified through a formal process evaluation, but rather reflect the lessons learnt from the experiences of an international team of GR experts. Moreover, some of the reported facilitators and barriers are not unique to the field of GR, but are in fact generalisable to all sorts of international studies in older persons.

The three most important methodological recommendations for conducting cross-national collaborative research in the field of GR are (1) make use of existing (inter)national networks and social media to aid recruitment of GR centres; (2) clearly define the GR treatment, setting, and patient characteristics in the inclusion criteria; and (3) use a hierarchical study structure to communicate transparently and regularly with both national and local coordinators using videocalls and SOPs.

The most important lesson learnt for the field of GR is the necessity to consistently and uniformly measure and register GR outcomes across different countries. International GR research is pivotal to optimising the quality of GR and would greatly benefit from the implementation of a core dataset in regular GR care. Therefore, future studies should focus on developing an international consensus regarding the outcomes and corresponding cross-culturally validated measurement instruments to be used during GR.

## Figures and Tables

**Table 1:** Facilitators and barriers for conducting cross-national GR research.

Facilitators	Barriers
**Recruitment of GR centres**	
Using regional, national, and international GR networks to promote study participation	Lack of tight regional and/or national GR networks
Choosing a research topic that is of interest to researchers in multiple countries	Heterogeneity in care setting and practice
Publishing protocol paper	Heterogeneity in eligible patients for GR
Appointing national coordinators who have a strong national network in GR and knowledge of national ethical regulations	Heterogeneity of legislation and procedures relating to ethical approval
Physical kick-off meeting for all national coordinators	
Posting recruitment calls on social media	
Transparency about co-authorship and data sharing policies	
Financial resources to conduct the study	
**Inclusion of GR patients**	
Appointing local coordinators to coordinate and promote the study within their centre	Main reason for referral can be ambiguous for multimorbid patients
Start-up videocalls with local coordinators	Patients may speak different languages
Regular (e.g., monthly) videocalls with national and local coordinators during inclusion period	Absence of back-up representatives for the local coordinators
Well-illustrated patient information sheet, i.e., comprehensible for older people	
Financial recourses for translation of consent forms and patient information sheets to local lay terms and minority languages	
**Data collection**	
Using regular care data from EHR ^1^, i.e., no additional measurements needed	Regular care data differs between countries, i.e., lack of core dataset
Standard Operation Procedures (SOPs) including step-by-step instructions for data collection	Heterogeneity in EHR ^1^ systems preventing automated data extraction
GDPR ^2^/HIPAA ^3^ compliant data capturing system	Lack of sensitive/specific cross-culturally validated questionnaires for GR
Drafting and regularly updating a data management plan and a metadata file.	

^1^ EHR = electronic health records; ^2^ GDPR = General Data Protection Regulation (EU); ^3^ HIPAA = Health Insurance Portability and Accountability Act (US).

## Data Availability

Not applicable.

## Supplementary Materials

The following are available online at https://www.mdpi.com/article/10.3390/jcm12030951/s1, Figure S1. Data sharing policy of the EU-COGER study.
