# Supplementary material for: How to Conduct International Geriatric Rehabilitation Research?
Source: J Clin Med. 2023 Jan 26;12(3):951. doi: 10.3390/jcm12030951 (PMC9917925; doi:10.3390/jcm12030951)
Supplement: Supplementary file 1 [file jcm-12-00951-s001.zip › jcm-2139789-supplementary.pdf]

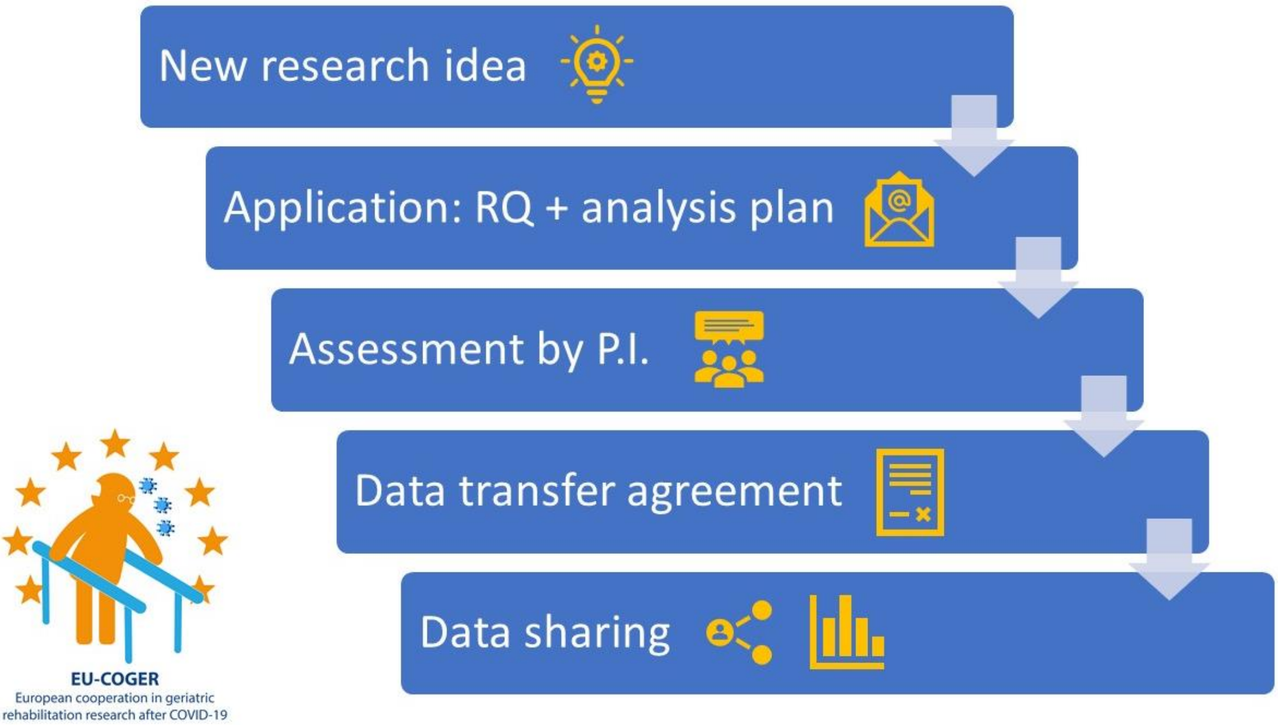

EU-COGER
European cooperation in geriatric rehabilitation research after COVID-19

Figure S1. Data sharing policy of the EU-COGER study. All persons with a new research idea, who wish to conduct additional analyses using EU-COGER data, may submit an application—including their research question (RQ) and analysis plan—to the principal investigator (PI). The PI will assess the novelty and feasibility of the application and upon approval a data transfer agreement must be signed by both parties, before data sharing can take place.
